# Distinct germ-line genetic mutation patterns correlate with reproductive outcomes in ICSI patients: a pilot study

**DOI:** 10.3389/fgene.2025.1610943

**Published:** 2025-05-23

**Authors:** Ting Jiang, Yan Wang, Wandai Wu, Qianru Yang, Sixian Wu, Xueguang Zhang, Wenming Xu

**Affiliations:** ^1^ Department of Obstetrics/Gynecology, Key Laboratory of Obstetric, Gynecologic and Pediatric Diseases and Birth Defects of Ministry of Education, West China Second University Hospital, Sichuan University, Chengdu, China; ^2^ Department of Obstetrics/Gynecology, Center of Reproductive Medicine, West China Second University Hospital, Sichuan University, Chengdu, China

**Keywords:** sperm whole-exome sequencing, ICSI outcomes, genetic variants, fertilization failure, implantation failure, pregnancy loss

## Abstract

**Background:**

Infertility affects approximately 15% of couples worldwide, with male factors accounting for nearly 50% of cases. Intracytoplasmic sperm injection (ICSI) has become the standard treatment for male factor infertility, but outcomes vary significantly among couples. While conventional genetic testing using blood samples is common in reproductive medicine, the genetic composition of sperm may differ significantly from somatic cells due to mosaicism and *de novo* mutations during spermatogenesis.

**Methods:**

We collected semen samples from 11 couples with varying ICSI outcomes: successful clinical pregnancy (n = 6), implantation failure (n = 3), and early pregnancy loss (n = 2). Sperm DNA was extracted using magnetic-activated cell separation and whole-exome sequencing was performed. The sequencing data were aligned to the GRCh37/hg19 reference genome and analyzed for potentially pathogenic mutations. Semen analysis and karyotype were also evaluated.

**Results:**

Semen analysis showed no significant differences between groups except for sperm morphology. Whole-exome sequencing identified distinct mutation patterns between groups. Mutations in *USP9X*, *SPAG6* and *ADGRG2* were observed in the clinical pregnancy group. Implantation failure and pregnancy loss were associated with mutations in genes involved in embryo adhesion, immune regulation, and genomic stability, including *MAGEC1*, *MUC4* and *SERPINA2*.

**Conclusion:**

This pilot study suggests that direct sperm exome sequencing may reveal genetic variants associated with different ICSI outcomes. While our findings require validation in larger cohorts, they generate hypotheses about sperm-specific factors that might influence post-fertilization developmental events and pregnancy outcomes.

## Introduction

Infertility is clinically defined as a couple’s inability to achieve conception after 1 year of regular, unprotected sexual intercourse (Zegers-Hochschild et al., 2009). It affects approximately 15% of couples worldwide, with male factor infertility accounting for nearly 50% of these cases (Cui, 2010). Since its introduction in 1991, intracytoplasmic sperm injection (ICSI) has revolutionized the treatment of male factor infertility by directly injecting a single spermatozoon into the oocyte cytoplasm, thereby circumventing natural fertilization barriers (Palermo et al., 1992; Van Steirteghem A. et al., 1993; Van Steirteghem A. C. et al., 1993). Despite the technical success of ICSI in achieving fertilization, significant variation exists in downstream outcomes including implantation, early embryonic development, and pregnancy maintenance. Even when fertilization occurs successfully, as evidenced by the formation of two pronuclei (2PN), embryo development may arrest or implantation may fail for reasons that remain poorly understood (O'Neill et al., 2018; Li et al., 2018). While female factors have been extensively studied in these contexts, the contribution of paternal genetic factors to post-fertilization outcomes deserves further investigation. (van den Hoven et al., 2015).

Over time, most genetic testing in reproductive medicines conducted using peripheral blood samples. However, this approach has critical limitations in the context of male fertility. Blood-derived DNA represents the somatic genome and may not accurately reflect the genetic composition of the male germline due to several key factors: (1) mosaicism specific to germline cells can create genetic diversity among sperm that is not present in somatic cells; (2) during spermatogenesis, *de novo* mutations may arise that would not be detected in blood samples; and (3) sperm-specific genetic alterations might remain undetectable when analyzing somatic cellsWhat is truly passed on to offspring are the genetic variants present in sperm, which may differ significantly from those detected in blood samples (Yang et al., 2021).

Advances in assisted reproductive technology (ART) and genomics have enabled direct examination of the sperm genome. Whole exome sequencing of sperm DNA, rather than blood DNA, can potentially detect gene mutations and single nucleotide variants (SNVs) unique to the germline, identifying factors affecting reproductive outcomes that would be missed by conventional genetic testing approaches (Ishida et al., 2025; Uddin et al., 2024; Oud et al., 2024). Yet most studies have focused on known infertility-related genetic conditions, while the contribution of sperm-specific variants to different ICSI outcomes remains largely unexplored (Xue et al., 2022).

Despite the availability of ancillary assessments and genetic testing, the sperm-related factors contributing to variable ICSI outcomes in cases with normal semen parameters remain elusive. We hypothesized that spermatozoa from men with different ICSI outcomes might harbor detectable genetic variations that could underlie these outcomes. To investigate the correlation between ICSI outcomes and male genetic factors, we collected semen samples from 11 men representing three distinct ICSI outcome groups (successful clinical pregnancy, implantation failure, and pregnancy loss), extracted DNA directly from purified sperm cells, and conducted whole-exome sequencing. By analyzing the germline genetic variants in these different outcome groups, we aimed to generate hypotheses about potential genetic factors influencing ICSI success rates and provide direction for future larger-scale studies.

## Materials and methods

### Study population and participants

This exploratory pilot study included 11 couples who underwent ICSI treatment at West China Second University Hospital, Sichuan University, from September 2022 to April 2023. Based on their ICSI outcomes, participants were classified into three groups: clinical pregnancy (n = 6), implantation failure (n = 3), and pregnancy loss (n = 2). Clinical pregnancy was defined as the presence of a gestational sac with fetal heartbeat at 6–7 weeks of gestation. Implantation failure was defined as the absence of implantation following the transfer of morphologically good-quality embryos in at least two consecutive ICSI cycles. Pregnancy loss was defined as spontaneous miscarriage after ultrasound confirmation of implantation. To minimize confounding factors, we applied the following inclusion criteria: (1) female Female partners were younger than 39 years with normal ovarian reserve (AMH >1.1 ng/mL) and normal ovarian response. Male partners with normal semen parameters and normal peripheral karyotype (46,XY); (3) no history of recurrent miscarriage, uterine abnormalities, or thrombophilia in female partners; and (4) absence of Y chromosome microdeletions in male partners. Written informed consent was obtained from all participants.

### The sperm samples

The collection and analysis of semen samples strictly followed the standards outlined in the WHO Laboratory Manual for the Examination and Processing of Human Semen. Sperm samples were collected from 11 male participants who ejaculated into a sterile container after 2–7 days of sexual abstinence. The samples were incubated in a 37°C water bath for 30–60 min to allow complete liquefaction before analysis. A computer-assisted sperm analysis (CASA) system was used to evaluate basic semen parameters. The following parameters were assessed: sperm concentration, semen volume, sperm morphology, sperm motility [Progressive motility rate (PR), Non-progressive motility (NP), Immotile sperm (IM)], ejaculate volume, and pH.

### Sperm DNA extraction and quality control

Sperm DNA was extracted from 1 mL of whole semen using a two-step process. First, motile spermatozoa were isolated using a density gradient centrifugation method to minimize somatic cell contamination. This was followed by magnetic-activated cell separation (MACS) to further purify the sperm population. Isolated sperm cells underwent lysis and DNA extraction using a modified protocol optimized for sperm chromatin decondensation (Qiagen Genomic DNA kit with dithiothreitol pretreatment). DNA purity was assessed using spectrophotometry (260/280 ratio >1.8) and integrity was verified using gel electrophoresis.

### Library preparation and sequencing

DNA samples that passed quality control were subjected to whole exome sequencing. The DNA was fragmented to approximately 400 bp using sonication, and exome capture was performed using the Agilent SureSelect Human All Exon V7 kit. Individual indexed libraries were constructed for each sample following the manufacturer’s protocol. Paired-end sequencing (2 × 150 bp) was performed on the Illumina NovaSeq 6000 platform, with a target mean coverage depth of 100× to ensure adequate detection of low-frequency variants that might be present in a subset of sperm cells.

### Bioinformatic analysis

The sequencing data were aligned to GRCh37/hg19 reference genome (map reads to reference) by GTX.Digest (Changsha, China). Mutation gene were filtered with the following criteria: Gene Quality (GQ) >20; SIFT score ≤0.05; PolyPhen2 score ≥0.447; MutationTaster prediction of 'Disease-Causing’; CADD score >15; and maximum minor allele frequency (MaxMAF) <0.05 in population databases (gnomAD, 1000 Genomes).

## Results

### Clinical characteristics of study participants

Our pilot study included 11 couples experiencing different ICSI outcomes: successful clinical pregnancy (n = 6), implantation failure (n = 3), and pregnancy loss (n = 2). All male participants had normal peripheral blood karyotypes (46,XY) and no Y chromosome AZF microdeletions. The clinical characteristics of all participants are summarized in Table 1. When comparing baseline clinical characteristics between groups, we found that male BMI was significantly higher in the implantation failure group (25.51 ± 0.73) compared to the clinical pregnancy group (20.88 ± 2.49; *p* = 0.0182). All other baseline clinical indicators, including female partner age, BMI, and hormonal parameters (AMH, FSH, E2), showed no statistically significant differences between groups (*p* > 0.05). Additionally, the couples underwent semen analysis testing (Table 2). The mean sperm concentration was 63.91 ± 21.03 million/mL, the mean percentage of normal sperm morphology was 9.11% ± 5.64%, the progressive motility rate was 51.36% ± 7.03%, and the non-progressive motility rate was 19.82% ± 2.44%, respectively, and the average semen volume among donors was 2.33 mL. The normal sperm morphology in the pregnancy loss group (3.45 ± 0.07) was significantly lower than that in the clinical pregnancy group (9.55 ± 4.52), with a *p*-value of 0.0354. Other semen parameters, including concentration, volume, and motility, did not differ significantly between groups.

**TABLE 1:** Baseline characteristics of fertile and infertile cohorts at study inclusion.

		Female age	Male age	Female BMI	Male BMI	Basal AMH level (ng/mL)	Basal FSH level (IU/L)	Basal E2 level (pmol/L)
	Couples (N = 11)	31.18 ± 4.51	33.27 ± 4.45	20.99 ± 2.28	22.55 ± 2.80	4.04 ± 3.09	5.66 ± 2.03	173.4 ± 343.1
Fertility group	Clinical pregnancy (N = 6)	29.17 ± 3.71	31.67 ± 1.75	20.11 ± 2.01	20.88 ± 2.49	4.54 ± 4. 02	5.79 ± 2.37	245.3 ± 462.2
Infertility group	Implantation Failure (N = 3)	33.00 ± 4.36	34.33 ± 6.43	22.41 ± 3.19	25.51 ± 0.73	2.45 ± 1.24	4.83 ± 2.16	122.4 ± 125.7
		*p* = 0.2078	*p* = 0.3471	*p* = 0.2176	*p* = 0.0182	*p* = 0.4225	*p* = 0.5753	*p* = 0.6762
	Pregnancy Loss (N = 2)	34.50 ± 6.36	36.50 ± 7.78	21.52 ± 0.22	23.14 ± 1.66	4.92 ± 0.85	6.51 ± 0.58	34.00 ± 5.66
		*p* = 0.1768	*p* = 0.1469	*p* = 0.3846	*p* = 0.2866	*p* = 0.9029	*p* = 0.7008	*p* = 0.5622

Note: A significant difference *p* < 0.05, Student’s t-test

**TABLE 2:** Semen routine parameters of fertile and infertile cohorts at study inclusion. PR rate: progressive motility rate. NP: non-progressive motility rate. IM rate: immotile sperm rate.

		Sperm concentration (million/mL)	Normal sperm morphology (%)	PR rate (%)	NP rate (%)	IM rate (%)	Ejaculate volume (mL)
	Couples (N = 11)	63.91 ± 21.03	9.11 ± 5.64	51.36 ± 7.03	19.82 ± 2.44	28.82 ± 5.85	2.33 ± 0.69
Fertility group	Clinical pregnancy (N = 6)	75.17 ± 17.19	9.55 ± 4.52	52.50 ± 6.98	19.00 ± 2.00	28.50 ± 6.29	2.48 ± 0.92
Infertility group	Implantation Failure (N = 3)	59.67 ± 11.50	12.00 ± 7.94	54.33 ± 5.13	20.33 ± 1.53	25.33 ± 3.79	2.13 ± 0.23
		*p* = 0.8462	*p* = 0.2072	*p* = 0.7021	*p* = 0.3486	*p* = 0.4566	*p* = 0.5502
	Pregnancy Loss (N = 2)	36.50 ± 19.09	3.45 ± 0.07	43.50 ± 6.36	21.50 ± 4.96	35.00 ± 1.41	2.15 ± 0.21
		*p* = 0.2348	*p* = 0.0354	*p* = 0.1602	*p* = 0.3038	*p* = 0.2166	*p* = 0.6465

Note: A significant difference *p* < 0.05, Student’s t-test

### Comparison of ICSI clinical outcomes among clinical pregnancy, implantation failure, and pregnancy loss groups

A systematic comparison of ICSI clinical characteristics was conducted among the three groups to investigate the differences in fertilization efficiency, embryonic development competence, and final pregnancy outcomes (Table 3). To assess embryonic development, various key percentage indicators were calculated. All percentages are based on the number of oocytes retrieved during each ICSI cycle, in accordance with standard clinical practice.

**TABLE 3:** Comparison of ICSI clinical outcomes among each patient group.

	Clinical pregnancy	Implantation failure	Pregnancy loss
Couples	6	3	2
Number of oocytes retrieved	77	31	17
Number of ICSI cycles	6	6	2
Number of MII oocytes (%)	57 (74%)	23 (74%)	11 (65%)
Number of 2PN (%)	39 (51%)	18 (58%)	7 (41%)
Number of 8-cells (%)	35 (45%)	14 (45%)	7 (41%)
Number of usable blastocysts (%)	14 (18%)	5 (16%)	2 (12%)
Number of transfer cycles	9	5	2
Number of embryos transferred per cycle	1	1	1
Implantation rate (%)	100%	0%	0%
Clinical pregnancy rate (%)	100%	0%	0%
Miscarriage rate (%)	0%	100%	100%

Note: All percentages are calculated relative to the number of oocytes retrieved in each cycle, which is the standard denominator used in clinical practice for assessing embryonic development.

In the Clinical Pregnancy group, a total of 77 oocytes were retrieved from 6 ICSI cycles, with 74% maturing to the MII stage. The fertilization rate, indicated by 2PN formation, reached 51%, and 45% of embryos developed to the 8-cell stage. Ultimately, 18% high-quality blastocysts were obtained, resulting in successful clinical pregnancies across nine transfer cycles.

In contrast, the Implantation Failure group showed comparable oocyte maturation (MII oocytes 74%) but a slightly higher fertilization rate (58%) compared to the Clinical Pregnancy group. However, the rates of 8-cell embryos (45%) and usable blastocysts (16%) were marginally lower. Despite five embryo transfer attempts, no successful implantation was achieved, suggesting possible defects in embryo competence or endometrial receptivity.

Notably, the Pregnancy Loss group demonstrated the lowest fertilization efficiency and embryo developmental potential. Among 17 retrieved oocytes from 2 ICSI cycles, only 65% reached the MII stage, and the fertilization rate dropped to 41%. Similarly, 41% of embryos developed to the 8-cell stage, and only 12% formed usable blastocysts. Although embryo transfer was achieved in both cycles, these pregnancies resulted in early pregnancy loss, indicating compromised embryo viability potentially associated with paternal genetic factors.

Collectively, these findings suggest that while oocyte maturation rates were relatively comparable across all groups, patients in the Implantation Failure and Pregnancy Loss groups exhibited reduced blastocyst formation rates and poorer clinical outcomes compared to the Clinical Pregnancy group. These observations highlight the possibility that paternal genetic defects may impair fertilization capacity, early embryonic development, or post-implantation viability, contributing to adverse reproductive outcomes following ICSI.

## Results of SNV analysis in semen donors

To investigate the impact of sperm genetic factors on assisted reproductive outcomes, we performed whole-exome sequencing (WES) on our cohort. Additionally, we conducted a comprehensive analysis of single nucleotide variations (SNVs) for each individual, aiming to identify potential genetic alterations associated with reproductive success or failure (Supplementary Material, Supplementary Table S1). This approach allows us to explore the contribution of inherited genomic variations to fertilization, embryo development, and pregnancy outcomes in assisted reproductive technologies.

Several genetic mutations identified in the clinical pregnancy group may have contributed to successful fertilization and embryo development despite prior infertility. For example, a previous study demonstrated that germ cell-specific knockout of *Usp9x* in mice resulted in normal development of spermatogonia during the early postnatal period. However, from the second postnatal week onward, *Usp9x*-deficient spermatogenic cells exhibited marked apoptosis at the early spermatocyte stage, ultimately leading to complete male infertility. These findings indicate that USP9X is essential for the mitosis-to-meiosis transition and/or the maintenance of early meiotic progression during spermatogenesis (Kishi et al., 2017).

Male infertility can result from impaired sperm motility due to multiple morphological abnormalities of the flagella (MMAF), which are frequently associated with structural defects in the axoneme. The radial spokes and other accessory structures encircling the central pair of microtubules are critical for generating the bending forces required for sperm motility. Previous studies have shown that mutations in *SPAG6* can lead to primary ciliary dyskinesia (PCD), a disorder characterized by defective motile cilia. Interestingly, during ICSI procedures, morphologically normal and motile spermatozoa have been retrieved from most PCD patients carrying SPAG6 variants, with the exception of patient P1. In our study cohort, although a potentially pathogenic SPAG6 variant was identified, no apparent defects in sperm morphology or motility were observed in the affected individual. This suggests that the pathogenicity of SPAG6 variants may be context-dependent or exhibit variable penetrance in terms of their impact on sperm flagellar function. (Liu et al., 2025; Bai et al., 2023; Xu et al., 2022). In addition, *ADGRG2* (a testis-specific adhesion G-protein-coupled receptor) (Kiyozumi & Ikawa, 2023; Hu et al., 2024) were identified in ICSI patients achieving clinical pregnancy. This suggests that while these mutations may contribute to male infertility (e.g., asthenozoospermia or impaired DNA repair), ICSI likely bypassed their functional deficits via direct sperm injection or maternal compensatory mechanisms.

These mutations potentially contribute to the primary infertility phenotype through distinct mechanisms (impaired DNA integrity, motility defects, or fertilization competence), yet their successful bypass via ICSI demonstrates: 1) Technical circumvention of sperm functional deficiencies through micromanipulation; 2) Possible maternal cytoplasmic compensation for paternal genetic lesions; 3) The existence of mutation-specific thresholds for developmental competence. Notably, the presence of mutations in non-essential genes (*MUC19*, ZNF family) further suggests genomic resilience in human reproduction. These findings underscore the need for functional characterization of paternal genetic contributions to early embryogenesis, particularly regarding mutation load and epigenetic reprogramming efficacy post-ICSI.

**FIGURE 1:**
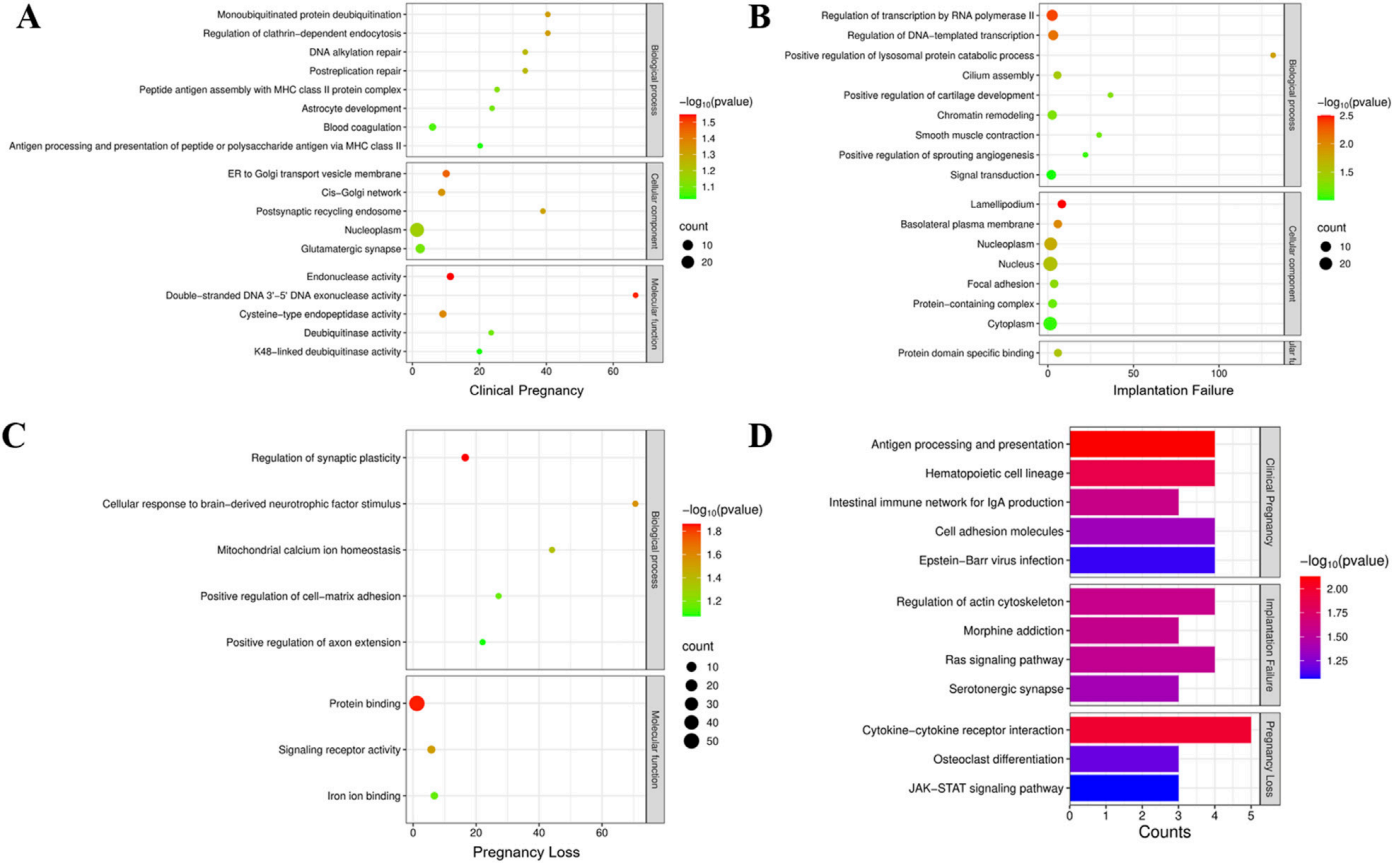
**(A–D)**: Gene Ontology (GO) enrichment analysis was performed to investigate the functional classification and biological processes associated with the mutation genes in clinical pregnancy group **(A)**, implantation failure group **(B)** and pregnancy loss group **(C)**. **(D)** Kyoto Encyclopedia of Genes and Genomes (KEGG) pathway enrichment analysis in each patient group.

Interestingly, GO enrichment analysis of the mutated genes identified in the Clinical Pregnancy group revealed significant enrichment in several biological processes, including mono-ubiquitinated protein deubiquitination, regulation of clathrin-dependent endocytosis, DNA alkylation repair, and postreplication repair (Figure 1A). These enriched pathways are closely related to cellular homeostasis, DNA damage response, and protein quality control, which are essential for maintaining sperm integrity and fertilization capacity. In addition, The KEGG pathway analysis of the Clinical Pregnancy group identified enriched pathways related to immune regulation and cell adhesion (Figure 1D). Mutations in genes associated with these pathways could affect immune tolerance, immune homeostasis, and embryo implantation, which are critical for successful pregnancy outcomes. These findings suggest that disruptions in immune function and cell adhesion may be tolerated regarding fertility and pregnancy success in ICSI patients.

In examining the genetic mutations associated with implantation failure and pregnancy loss, several overlapping mutations point to shared underlying biological mechanisms contributing to reproductive failure. Mutations in genes like *SERPINA2*, *SCAPER*, and *MAGEC1* appear in both groups, suggesting their critical roles in immune regulation, cell cycle progression, and genomic integrity (Table 3). Mutations in *SCAPER* have been identified across diverse populations and are associated with varying degrees of intellectual disability, retinitis pigmentosa, and ciliopathies (Tatour et al., 2017). SCAPER is known to regulate cyclin A2 localization and influence M-phase progression, suggesting that male infertility in *SCAPER*-deficient individuals may result from impaired cyclin A2 function (Tatour et al., 2017; Pagano et al., 1992; Chotiner et al., 2019; Tatour et al., 2020). Studies have demonstrated that patients homozygous for *SCAPER* mutations exhibit a complete loss of *SCAPER* expression in spermatogonia (SPG), leading to azoospermia due to early-stage spermatogenic failure and the absence of meiotic cells (Wormser et al., 2021). Further studies will be required to explore the specific pathways by which SCAPER fulfills its roles in these systems.

MAGEC1 belongs to the MAGE family of genes, which are exclusively expressed in tumors and male germline cells (Bode et al., 2014). These genes encode antigens recognized by T lymphocytes, making them potential targets for antitumor immunotherapy. Recurrent detection of *MAGEC1* mutations in patients with male factor infertility highlights its role in maintaining genetic stability during early embryonic development (Zhou et al., 2024). *SERPINA2* is classified as an atypical or pseudogene, and its precise function remains largely undefined (Seixas et al., 2007; Marques et al., 2013). However, it is noteworthy that SERPINA2 belongs to the serpin (serine protease inhibitor) superfamily, which plays a crucial role in regulating protease activity. Members of this family are primarily involved in physiological processes such as coagulation, fibrinolysis, immune response, and inflammation modulation, so SERPINA2 may disrupt immune responses or coagulation pathways, potentially hindering the proper implantation of the embryo or preventing the maintenance of pregnancy (Wang et al., 2024; Janciauskiene et al., 2024).

Nevertheless, distinct differences between the implantation failure and pregnancy loss groups indicate that reproductive failure arises at different stages of pregnancy, influenced by mutations specific to each process. In the implantation failure group, mutations in genes such as *MUC4* and *GPR35* suggest that issues related to cellular adhesion and immune system modulation might impair the embryo’s ability to successfully attach to the uterine lining. MUC4, a member of the mucin (MUC) family, belongs to a group of high-molecular-weight glycoproteins that play essential roles in lubricating and protecting epithelial surfaces within the reproductive tract. MUC4 is crucial for cellular adhesion and protection, and its mutation may disrupt the interaction between the embryo and the endometrial lining, preventing successful implantation. In the context of unexplained recurrent pregnancy loss (uRPL), MUC4—marked by altered mucus secretion—has been identified as a key pathogenic factor (Lin et al., 2025; Lee et al., 2024; Kim et al., 2022). Similarly, *GPR35*, a G-protein coupled receptor, plays a role in immune modulation, and its mutation could interfere with the immune environment necessary for the embryo’s integration into the uterus (Shu et al., 2022; Wang et al., 2022). In contrast, the pregnancy loss group presents mutations in genes such as *TGFBR2* and *IFNAR2*, which are more involved in immune regulation and cellular stability after implantation (Hurtado-Guerrero et al., 2020). A prominent role for the BMP/TGF-β pathway in regulating the patterning of early embryos has been described (Cañón-Beltrán et al., 2024). Interferon-tau (IFNT), a type I interferon, is an antiluteolytic factor secreted by trophoderm during pregnancy. IFNT transmitted signals or stimulated the expression of some factors to build maternal recognition and keep pregnancy by binding its receptors, IFNT receptor 1(IFNAR1) and IFNT receptor 2 (IFNAR2) (Wang et al., 2018).

GO enrichment analysis of the mutated genes identified in the Implantation Failure group revealed significant enrichment in several critical biological processes (Figure 1B). These findings suggest that genetic mutations affecting transcriptional regulation and lysosomal protein degradation may disrupt early embryonic gene expression and cellular homeostasis, which are essential for successful embryo implantation. Furthermore, the enrichment of pathways related to cilium assembly indicates that mutations in genes critical for ciliary structure or function may impair sperm motility and hinder proper embryo-endometrial interactions, both of which are vital for successful implantation. In addition, KEGG pathway analysis of the group highlighted the enrichment of pathways involved in cell signaling and cytoskeletal dynamics (Figure 1D). These pathways are integral to trophoblast invasion and embryonic development, and their disruption could significantly affect the implantation process.

Similarly, for the Pregnancy Loss group, GO analysis revealed enrichment in biological processes associated with regulation of synaptic plasticity, cellular response to brain-derived neurotrophic factor (BDNF), and mitochondrial calcium ion homeostasis (Figure 1C). Given the pivotal roles of mitochondrial function, calcium signaling, and cellular energy metabolism in embryonic development and survival, mutations in these pathways are likely to compromise embryonic development, leading to an increased risk of pregnancy loss. Additionally, KEGG pathway analysis revealed enrichment in immune-related pathways, which suggest that immune dysregulation and abnormal tissue remodeling could contribute to pregnancy failure.

These mutations suggest that while implantation might initially occur, the subsequent loss of pregnancy could be attributed to immune rejection, improper cellular division, or metabolic failure during pregnancy development. Collectively, these differences emphasize the need to understand the specific timing and biological context of genetic mutations in reproductive failure, highlighting distinct mechanisms governing early implantation versus the sustained success of pregnancy.

## Discussion

This pilot study represents the first investigation comparing sperm-specific genetic variants across different ICSI outcomes, including successful clinical pregnancy, implantation failure, and early pregnancy loss. While our sample size is limited to 11 couples, our findings provide important preliminary insights into the potential role of paternal genetic factors in determining reproductive success following ICSI treatment. The identification of distinct genetic variant patterns associated with each outcome group suggests that sperm DNA analysis may offer valuable information beyond conventional semen parameters and blood-based genetic testing.

The distinctive pattern of genetic mutations observed in each reproductive outcome group—clinical pregnancy, implantation failure, and pregnancy loss—provides critical insights into the molecular basis of ICSI success and failure. Our analysis revealed that while certain mutations (*USP9X*, *SPAG6*, *ADGRG2*) may impair natural fertility, they can be effectively bypassed through ICSI, likely due to the technique’s ability to circumvent functional limitations of the sperm or through maternal compensatory mechanisms. This finding aligns with the clinical observation that ICSI can achieve high fertilization rates even in cases of severe male factor infertility, particularly those with motility issues related to flagellar defects.

Conversely, the mutations identified in the implantation failure and pregnancy loss groups primarily affect processes that occur post-fertilization, such as embryo-endometrial interaction (*MUC4*, *GPR35*), immune tolerance (*SERPINA2*), and cell cycle regulation (*SCAPER*). These processes cannot be bypassed simply through ICSI, explaining why these patients experienced reproductive failure despite successful fertilization. This distinction highlights the need for differentiated approaches to infertility treatment based on the underlying genetic etiology, particularly in cases of recurrent ICSI failure.

The detection of genetic mosaicism in sperm samples—where only a proportion of spermatozoa carry deleterious mutations—offers a potential therapeutic strategy for patients with recurrent ICSI failure. Our findings suggest that fertilization outcomes might be improved by either increasing the number of fertilization attempts to enhance the probability of selecting mutation-free sperm or by developing methods to selectively isolate genetically competent spermatozoa. Further research into techniques for identifying and selecting genetically intact sperm cells could significantly advance the treatment options for patients with otherwise unexplained fertilization failure.

Our study has several limitations that warrant consideration. The small sample size limits the statistical power and generalizability of our findings. Additionally, while we identified potential pathogenic mutations, functional validation studies are needed to establish definitive causal relationships between these genetic variants and the observed reproductive outcomes. Finally, our analysis focused primarily on protein-coding regions, potentially missing relevant variations in non-coding regulatory regions that might influence fertility. Future research should aim to expand sample sizes, include functional validation of identified mutations, and incorporate analysis of non-coding regions. Longitudinal studies following patients through multiple treatment cycles would provide valuable insights into the consistency and predictive value of sperm genetic profiles for reproductive outcomes. Finally, developing targeted gene panels based on our findings could offer a more cost-effective screening approach for clinical implementation.

## Conclusion

In conclusion, this pilot study provides preliminary evidence that direct genetic analysis of spermatozoa can reveal valuable information about potential ICSI outcomes that is not captured by conventional semen analysis or blood-based genetic testing. While our findings require validation in larger cohorts, they suggest that different genetic mechanisms may underlie various forms of reproductive failure following ICSI. The distinct patterns of variants associated with clinical pregnancy versus implantation failure and pregnancy loss highlight the complexity of genetic factors influencing reproductive success and suggest that personalized therapeutic approaches based on sperm genetic profiles may improve outcomes for couples struggling with infertility. As we advance toward precision medicine in reproductive health, comprehensive genetic evaluation of both maternal and paternal contributions will be essential for optimizing treatment strategies and improving success rates in assisted reproduction.

## Data Availability

The authors acknowledge that the data presented in this study must be deposited and made publicly available in an acceptable repository, prior to publication. Frontiers cannot accept a manuscript that does not adhere to our open data policies.
