# Supplementary material for: Distinct germ-line genetic mutation patterns correlate with reproductive outcomes in ICSI patients: a pilot study
Source: Front Genet. 2025 May 23;16:1610943. doi: 10.3389/fgene.2025.1610943 (PMC12141242; doi:10.3389/fgene.2025.1610943)
Supplement: Supplementary file 1 [file Supplementaryfile1.docx]

**Supplementary information, Table S1. List of Genes with Mutations Identified in Each Patient Group**

| **Clinical Pregnancy** | | | | | | **Implantation Failure** | | | **Pregnancy Loss** | |
| --- | --- | --- | --- | --- | --- | --- | --- | --- | --- | --- |
| **Patient 1** | **Patient 2** | **Patient 3** | **Patient 4** | **Patient 5** | **Patient 6** | **Patient 1** | **Patient 2** | **Patient 3** | **Patient 1** | **Patient 2** |
| PAPSS2 | SPRY4 | GRM1 | FADS6 | ADGRG2 | CCR9 | MUC4 | CFAP47 | POTED | TGFBR2 | DDX3Y |
| CES2 | MUC19 | PKIG | CDKAL1 | HLA-DQB1 | NPIPB11 | DAG1 | ARF1 | PDE10A | IGSF1 | BIVM |
| FERMT3 | PNPLA4 | LAMC1 | DOHH | MUC12 | KRTAP5-5 | GSN | C19orf12 | MYLK | VPS13B | ZBTB8A |
| ANGPTL6 | CDK6 | PRB1 | TSNARE1 | USP7 | NPIPA5 | GPR35 | ZNF789 | UGT2A1 | TRNT1 | ATRN |
| SIPA1L3 | LIPT1 | RAD9A | GOLGA8G | LAS1L | PLXNA3 | TRPS1 | COMMD5 | FGF1 | UNC45B | TCFL5 |
| COX6B2 | C14orf180 | TAF6 | ACTRT1 | ASXL1 | SERPINA2 | SATB1 | ARID4A | GGA3 | LIPT1 | GOLGA6L4 |
| SNAP91 | HNRNPH3 | GOLGA6L6 | GOLGA6L3 | CCDC106 | CYP2D7 | PER2 | CMTR2 | TBL3 | LZTS1 | GPR1 |
| C3orf35 | IQCM | SERPINA2 | PCDH11Y | RIMBP3B | CD8B | VRK2 | ZNF180 | GGTLC3 | IFNAR2 | ZNF18 |
| AKTIP | DCAKD | SKOR2 | GCNA | GOLGA8K | APEX2 | PAK4 | KIF4A | SPATA31C1 | FRS2 | ACR |
| USP9X | KRTAP5-5 | RBM23 | MAGEC1 | NPIPB11 | VBP1 | ASCC2 | PPP4R3C | MAGEC1 | ANXA6 | P4HA1 |
| CCDC32 | UBE2A | SLFN11 | KHDRBS3 | CTAGE4 | SUPT20HL1 | NPIPA5 | MAGEC1 | PDZD11 | CNTFR | GTDC1 |
| PDP2 | LOC101059915 | MUC19 | SPAG6 | PPHLN1 |  | ZC4H2 | SLC25A43 | TFDP3 | CNTNAP4 | SERPINA2 |
| CFAP94 | PNMA6E | ZNF630 | ZNF799 | FGF1 |  | LRP1 | CFAP94 |  | OPRL1 | CNR2 |
| ZNF532 | MAGEC1 | SHANK3 | UPK3B | ZNF333 |  | GPRIN2 | SCAPER |  | CDA | SAMD7 |
| TARM1 | GPRIN2 | CPVL | TRIM64 | LSM14A |  | SCAPER | SLC16A12 | | CCDC182 | PPIL3 |
| SLC16A7 | ARHGAP4 | SPARA31C1 | HMBS | C1QTNF7 |  | GABRB2 | ARIH2 |  | NBPF15 | INSYN2A |
| GSN | CD59 | USP11 | ZNF302 | P4HA1 |  | SERPINA2 | VGLL4 |  | ZNF107 | TRIM64 |
| TOP2B | COX17 | REPS2 | DGKK | TBL3 |  | MRLN | SERPINA2 | | KRTAP5-5 | P2RY2 |
| PAFAH1B2 | ZNF183 |  | GRIPAP1 | ADRA1A |  | ESF1 | MLLT6 |  | LGALS3BP | ATP6V0A1 |
| RTL4 | NBPF19 |  |  | HDX |  | ZNF707 | SOX6 |  | TMLHE | USP38 |
| LRRC4C | MUC4 |  |  |  |  | MCM3 | JAKMIP3 |  | EIF1AY | ZMYM3 |
| SLU7 | MED20 |  |  |  |  | SMAD1 | NBPF15 |  | MAGEC1 | UBR2 |
|  | STAU2 |  |  |  |  | KIF27 | CEACAM5 | | CCR6 |  |
|  | GOLGA6L2 | |  |  |  | GNG5 | CCDC57 |  | DISC1 |  |
|  | HLA-DRB5 | |  |  |  | IKBKB | GGTLC3 |  | SCYL2 |  |
|  | NFYC |  |  |  |  | NPIPB3 | APBB2 |  | CCL28 |  |
|  | GOLGA8S |  |  |  |  | CYP2D7 | SPATA31C1 | | SOCS1 |  |
|  | PIAS2 |  |  |  |  | TOP2B | ZXDA |  | KIF27 |  |
|  | DYDC1 |  |  |  |  | GDPD2 | LOC100133315 | | TBC1D5 |  |
|  | AP3M1 |  |  |  |  |  | DUSP19 |  | MED20 |  |
|  | HGC6.3 |  |  |  |  |  | ZNF521 |  | GALNT13 |  |
|  | ELL |  |  |  |  |  | TNRC6C |  | MAPT |  |
|  | TFDP3 |  |  |  |  |  |  |  | TMCO5A |  |
|  | SLU7 |  |  |  |  |  |  |  | STATH |  |
|  |  |  |  |  |  |  |  |  | CYP2D7 |  |
|  |  |  |  |  |  |  |  |  | UBE3A |  |
|  |  |  |  |  |  |  |  |  | KLHDC7B | |
|  |  |  |  |  |  |  |  |  | AMMECR1 | |
|  |  |  |  |  |  |  |  |  | PTGR1 |  |
|  |  |  |  |  |  |  |  |  | SUPT20HL1 | |
|  |  |  |  |  |  |  |  |  | NLGN4Y |  |
|  |  |  |  |  |  |  |  |  | LACC1 |  |
